# The epidemiology of college alcohol and gambling policies

**DOI:** 10.1186/1477-7517-2-1

**Published:** 2005-02-09

**Authors:** Howard J Shaffer, Anthony N Donato, Richard A LaBrie, Rachel C Kidman, Debi A LaPlante

**Affiliations:** 1 Harvard Medical School, Division on Addictions, The Landmark Center, 401 Park Drive, 2nd Floor East, Boston, MA 02215, USA

## Abstract

**Background:**

This article reports the first national assessment of patterns of drinking and gambling-related rulemaking on college campuses (e.g., punitive versus recovery oriented). Analyses relating school policies to known school rates of drinking or gambling identified potentially influential policies. These results can inform and encourage the development of guidelines, or "best practices," upon which schools can base future policy.

**Methods:**

The college policy information was collected from handbooks, Web sites and supplemental materials of 119 scientifically selected colleges included in the fourth (2001) Harvard School of Public Health College Alcohol Study (CAS). A coding instrument of 40 items measured the scope and focus of school alcohol and gambling policies. This instrument included items to measure the presence of specific policies and establish whether the policies were punitive or rehabilitative. A total of 11 coders followed a process of information extraction, coding and arbitration used successfully in other published studies to codify policy information.

**Results:**

Although all schools had a student alcohol use policy, only 26 schools (22%) had a gambling policy. Punitive and restrictive alcohol policies were most prevalent; recovery-oriented policies were present at fewer than 30% of schools. Certain alcohol and gambling policies had significant relationships with student binge drinking rates.

**Conclusions:**

The relative lack of college recovery-oriented policies suggests that schools might be overlooking the value of rehabilitative measures in reducing addictive behaviors among students. Since there are few college gambling-related policies, schools might be missing an opportunity to inform students about the dangers of excessive gambling.

## Background

Young people are at increased risk for alcohol- and gambling-related problems compared to their older counterparts [1-3]. College and university students are at special risk because going to college often represents the first move away from their family and, as a result, fewer restrictions on their activities. (Because universities are by definition comprised of colleges, all institutions of higher learning henceforth will be referred to as "colleges.") In the United States, each year approximately 1.2 million freshmen enter four-year colleges [4]. Some of these freshmen enter college actively involved in recovery programs for alcohol abuse or other addictive behaviors (e.g., illicit drug abuse or gambling). Others will begin a program of recovery for addiction problems that started after they enrolled at school. The college years are a time of developmental transition for most students; like other life transitions, the college experience can be associated with increased risk for a variety of psychosocial problems.

The problems associated with addictive behaviors on college campuses have been well documented (e.g., academic difficulties, psychosocial problems, traumatic injuries, overdoses, high-risk sexual behavior, and impaired driving) (e.g., Wechsler et al. 2000 [5], Wechsler et al. 2002 [6]). Despite a recent increase in college-based preventative measures (e.g., alcohol education programs, advertising restrictions, alcohol-free dormitories, policy controls), research reveals that addiction-related problems continue to plague college campuses. For example, during the past decade, past-year alcohol use and binge drinking rates have remained steady at approximately 81% and 44%, respectively [6], and alcohol-related problems have been on the rise. Wechsler et al. (2002 [6]) found that a greater percentage of students who had used alcohol in the past 30 days were involved in police-related incidents in 2001 than in 1993 (6.5% vs. 4.6%); the same was true of alcohol-related injuries (12.8% vs. 9.3%). Wechsler et al. (2002) also identified a significant increase in the rate of students riding in motor vehicles with alcohol-impaired drivers in 2001 compared to 1993 (23.2% vs. 18.4%). These findings highlight the need for college administrators to reconsider current preventative measures and develop and implement more effective methods for preventing and reducing alcohol use. For example, college health programs might be able to limit or reduce alcohol-related harms on college campuses by implementing and enforcing policies that support recovery-oriented and other programs that discourage substance misuse.

The creation and implementation of college alcohol and gambling policies is far from an exact science. Currently, there are no standardized scientific guidelines for the creation of school policy directed toward alcohol and other potentially addictive behaviors (e.g., gambling). However, science can contribute to the creation of successful policy. Recognizing the important role that science can play in the development and evaluation of public policy, the federal government recently released draft "regulatory science" guidelines [7]. The Office of Management and Budget (OMB) intends these guidelines to direct and inform public agencies in the creation and implementation of effective and targeted regulations. Science-based guidelines also could prove useful to policymaking on college campuses; however, as the results of this study will reveal, college administrators do not use empirical evidence to guide the development and implementation of student substance use and gambling regulations. This situation has led to disjunctive policy strategies among U.S. colleges.

The purpose of this study is to encourage the development of science guided school policy. To accomplish this goal, we will examine the prevalence and characteristics of alcohol- and gambling-related policies, including policy provisions for student recovery, in a scientifically selected sample of U.S. colleges. We will not include illicit drug policies in this analysis because illicit drug use is illegal for both adults and young people; these illegal behaviors fall under the purview of state and federal law that supersedes college policy. Our intent is to examine college policies that focus on legal activities. Therefore, using college alcohol and gambling policies, binge drinking rates and gambling frequency as evidence, this report describes the epidemiology (e.g., prevalence) and influence of these assorted policies.

### Filling the Policy Void: A Federal Drug and Alcohol Initiative

During 1989, the federal government initiated basic alcohol and substance abuse education requirements. Previously, there was not a regulatory mandate obligating institutions of higher learning to set alcohol or drug use policy or bring students' attention to these rules if they existed. Schools also were not required to disseminate substance use policy information to parents or other interested parties. This situation changed with the passage of the federal Drug-Free Schools and Communities Act (DFSCA) of 1989.

The DFSCA applies to all U.S. colleges. The act specifies that "as a condition of receiving funds or any other form of financial assistance under any Federal program, an institution of higher education (IHE) must certify that it has adopted and implemented a drug [and alcohol] prevention program..."[8]. Thus, any U.S. college that does not maintain a drug and alcohol education program risks losing all of its federal funding. In addition, to fulfil DFSCA requirements and retain funding, schools must provide students with institutional standards of conduct that explicitly prohibit illicit drugs and illegal alcohol use, a description of potential legal and institutional sanctions for substance use violations, a description of health risks posed by drugs and alcohol, and a listing of available treatment options.

### The Impact of Government Policy on College Campus Substance Use and Abuse is Unknown

The overall impact of mandated drug and alcohol programs is still unknown; as we noted before, there is some evidence that risky and addictive behaviors on college campuses are still prevalent despite targeted efforts by administrators to reduce student substance abuse [9,6]. Several studies have suggested that, despite prevention efforts, established norms of excessive drinking behavior and positive student attitudes regarding the effects of alcohol consumption continue to encourage alcohol consumption on college campuses [10,11]. The absence of universal standards governing the content of school policies on addiction might contribute to this problem. Although the DFSCA mandates that schools must make written drug and alcohol policy available to students on an annual basis, administrators at each institution still determine the content of such policy. Thus, the DFSCA mandates policy without establishing standards for content; as a result, administrative tolerance toward alcohol, drugs, and gambling can vary significantly from institution to institution.

### The Potential Effect of Inconsistent College Policies

Inconsistent policy content among institutions can create a problematic state of affairs. Although DFSCA directives aim to increase awareness of the potential dangers of alcohol and drug use among students, numerous studies continue to identify high levels of alcohol abuse on U.S. college campuses in recent years [9,12-14,6]. Heavy episodic drinking adversely affects not only those students who actively participate, but also those who do not: one study identified non-heavy drinkers on heavy drinking campuses as 3.6 times as likely to experience at least one problem from another student's drinking as non-heavy drinkers on non-heavy drinking campuses [15].

Even though individual colleges have adopted different strategies for reducing the problems associated with excessive alcohol consumption, the extent and effect of these efforts are largely unknown. One approach, perhaps in response to DFSCA, has been to develop and enforce policies on student substance abuse and recovery. Although recent psychosocial programs attempting to reduce student drinking behaviors have failed to reduce binge drinking [6], official school policies on substance abuse and recovery hold the potential to reduce students' alcohol use and the multitude of consequential problems associated with drinking excessively. This potential, however, is likely contingent upon policy content: because there are few federal regulations governing the content of alcohol policies at institutions of higher learning, every college develops unique strategies of combating potentially addictive behaviors. To date, no studies have examined the policy content of a representative sample of colleges in the attempt to identify the effects of these policies on levels of alcohol and gambling involvement among students.

### Policy and Recovery

Students who seek help for alcohol or other substance use problems are faced with a multitude of school-provided and external treatment options. Addiction recovery programs are diverse, ranging from formal treatment programs (e.g., inpatient medical treatment and outpatient psychotherapy) to less formal self-help options, (e.g., 12-step fellowships) [16]. Regardless of the selected type of treatment, attention to recovery from addiction requires significant time and determination, which can disrupt a schedule of college studies. Twelve-step programs, for example, usually involve attending regular, perhaps even daily meetings. Formal treatment programs frequently demand an even greater level of time commitment: in-patient detoxification or other residential care can remove students from the academic environment altogether. Mandatory abstinence, required by most treatment programs, poses an additional hurdle to treatment-seekers. Students, with their busy and often stressful schedules, undoubtedly face additional challenges in participating in recovery activities; academic and administrative policies that accommodate flexible scheduling will likely assist students seeking recovery, and policies that do not might complicate or inhibit students' recovery efforts.

### College Binge Drinking and School Policy

Binge drinking, the consumption of five or more alcoholic drinks (four or more for women) on at least one occasion at one to two week intervals [12], has been unaffected by prohibitive and punitive college policies. To illustrate, on one college campus that prohibited all alcohol use in its residence halls, there was virtually no difference in the binge drinking rate among students living within areas regulated by the alcohol policy (35%) compared to those living outside the jurisdiction of the alcohol policy (34%) [17]. Although school policy (or the lack thereof) is not the only factor that affects binge drinking rates – promotions aimed at students, cheap alcohol prices at surrounding establishments and high numbers of on- and off-campus drinking venues have been found to significantly increase student binge drinking [18] – placing special emphasis on the enforcement of substance abuse policies can garner positive results. For example, Knight (2003)[19] found that, although the effect of policy was diluted by considerable variation in policy content among public colleges in a state-wide system, increased *enforcement *(i.e., application of policy consequences) of alcohol policies aimed at combating underage drinking did result in decreased alcohol consumption among students. Beneficial effects resulting from the enforcement of existing rules, however, can be difficult to interpret. For example, in that study, it is unclear whether the enforcement of rules encouraged lower levels of drinking or entry to treatment for intemperate drinking or, alternatively, simply forced problematic drinkers to withdraw from school.

### College Gambling and School Policy

Some research suggests that gambling on college campuses is commonplace. A study of student gambling at six colleges in five different states (i.e., New York, New Jersey, Nevada, Oklahoma, and Texas) showed that of 1,771 surveyed students, 23% reported that they gambled at least weekly (ranging from 11% in Texas to 39% in Nevada) [20]. In that study, students reported whether they had ever experienced gambling-related problems as identified by the South Oaks Gambling Screen (SOGS) [21]. Of the total student sample, 5.5% were classified as lifetime probable pathological gamblers. The prevalence of lifetime pathological gamblers among these students ranged from 4% in Nevada to 8% in New York. A recent report [22] of a four-campus Connecticut college system reported a similar SOGS-based prevalence estimate of probable pathological gamblers (i.e., 5.2%).

For comparison, the National Gambling Impact Study Commission (NGISC) considered the adult rates of lifetime pathological gambling from four sources [3]. The lowest rates were 0.8% for both the University of Michigan [23] and National Opinion Research Center [24] studies; the largest (i.e., 1.5% – 1.6%) were from aggregated statistics of previously published research conducted by the National Research Council [25] and the original analysis of the same studies by the Harvard Medical School [26]. This meta-analysis included 14 SOGS-based studies of disordered gambling among college students and indicated that the lifetime prevalence of pathological gambling among college students was 5.1% [26]. An update of this meta-analysis expanded the number of student studies to 19 and increased the prevalence estimate to 5.6% with a 95% confidence interval of 3.5% to 7.6% [1]. Based on 66 studies of the general household population in various areas (i.e., states), this estimate of the proportion of college students with gambling disorders was three times the adult rate (1.9%).

Other research contradicts the findings that college students are at elevated risk for problem gambling compared to the general adult population. For example, a recently published longitudinal study of students at the University of Missouri-Columbia showed markedly lower prevalence rates than the studies summarized above [27]. In this longitudinal study, no student met the traditional criteria for problem or pathological gambling. Further, the authors note that, "there were too few participants endorsing multiple gambling problems at a single time point to obtain an adequate sample size of affected individuals for most analyses" (Slutske et al. 2003[27] p. 265). Overall, 3% of these students endorsed a single problem at any point during their lifetime due to gambling; one student endorsed two problems and all of the others reported never having had a problem due to gambling. At the next interview three years later, when most subjects were seniors, the subjects reported more symptoms; but only one subject (i.e., 0.2% of the sample) endorsed enough symptoms to meet the diagnostic criteria of the American Psychiatric Association [28] for lifetime pathological gambling. This evidence indicates that gambling behavior among students and its adverse consequences fluctuates with time and other factors and that the development of symptoms is not always progressive. Further, the Slutske results show that most adverse effects of student gambling remain sub-clinical, making this pattern more responsive to interventions than longer standing, more entrenched clinical disorders. Taken together, this evidence suggests that comprehensive college gambling policies might have the capacity to reduce the adverse consequences that can be associated with student gambling.

Despite the frequency with which college students engage in gambling activities, some evidence suggests that administrators are unaware of the dangers associated with excessive gambling among students; in addition, colleges do not have adequate policies addressing gambling [29]. This situation prompted Shaffer to suggest that the government convene "a consortium of college presidents to review their existing gambling related policies and problems so that we can take a systematic approach to the education, prevention and treatment of America's young people, who are at higher risk for gambling related disorders than their adult counterparts"[30]. Although this consortium has not yet been assembled, research confirms that college students continue to view gambling as a legitimate form of entertainment; for example, 42% of a scientifically selected sample having gambled at least once in the last year [31]. Unlike drug and alcohol education (i.e., DFSCA), there is no federal mandate requiring schools to educate students or parents about the dangers of excessive gambling; combined with the lack of a policy response by administrators, this situation leaves an open door for student-related gambling disorders to emerge unchecked.

### Assessing the Relationships between College Policies and Student Drinking and Gambling

This study is the first to identify patterns of drinking and gambling-related rulemaking on college campuses (e.g., punitive versus recovery oriented). By relating school policies to known school rates of drinking or gambling [31,6] we can identify potentially influential policies. These analyses can encourage and inform the development of guidelines, or "best practices," upon which schools can base future policy.

### Hypotheses

Given the paucity of empirical college-based policy research, this study will fill an important gap in knowledge. To fill this void, this research will test a variety of addiction-related hypotheses that have not yet been examined empirically. Based upon the extant literature, this study will test the following four primary hypotheses:

• Because there are few requirements guiding the creation of school substance use and gambling policies, the content and clarity of these policies will be heterogeneous across schools and modes of policy distribution (e.g., handbooks vs. school Web sites);

• College alcohol policies currently devote relatively little attention to student recovery;

• Due to differences in enforcement, awareness of the dangers of excessive alcohol consumption, educational programs and types of students, schools with either no or only *restrictive *alcohol use policies will experience higher levels of binge drinking among students than schools with *prohibitive and recovery-oriented *alcohol policies;

• Absent a federal mandate that requires gambling-related regulations or education on college campuses, gambling policies will be less prevalent than alcohol use policies.

## Methods

### Procedure: Sample, Policy Eligibility and Policy Selection

The purpose of this study is to identify and assess alcohol and gambling policies among U.S. colleges. To ensure a representative national sample of colleges, we examined the scientifically selected sample of public and private American colleges that was used in a recent series of Harvard studies (e.g., Wechsler 2002 [6]). The detailed methods by which the previous study identified the sample are available elsewhere [6,12-14,31]. The potential sample consisted of 120 scientifically selected schools located throughout the nation; one school ceased operation before the start of the study, so 119 schools were eligible to be included in the final sample. We received human subjects approval for this study through the Harvard Medical School Office for Research Subject Protection. On February 14, 2003, the Human Subjects Committee at Harvard Medical School granted an exemption for the study entitled: *United States College and University Addiction and Recovery Policies. *The study qualified for exemption under 46 CFR §102(f) and the assurance identification number is M1240-01.

At the beginning of the project, we submitted an e-mail request for a hard copy of their student handbook to each school's admissions office. Each e-mail specified that we were interested in collecting school alcohol and gambling policies and requested that our inquiry be forwarded to the most appropriate school official. We gathered e-mail addresses for admissions offices from each school's official Web site. Using each school's main telephone number to initiate contact, investigators contacted schools that did not respond within thirty days to our e-mail request and verbally requested a handbook and any other existing alcohol and gambling policy materials. Typically, the person answering the call referred us to admissions offices, deans' offices, or student services offices for further assistance; we identified ourselves as calling from Harvard Medical School only when asked.

### Policy Eligibility and Identification

#### Eligibility Criteria

To be eligible for inclusion in this study, each college policy had to meet the following five eligibility criteria:

1. the policy had to prohibit, govern, or otherwise attempt to regulate alcohol use or gambling among students at a U.S. college or university;

2. the policy had to be in effect (i.e., in the current handbook, Web site or supplementary materials);

3. the policy had to be readily available to the public, either in electronic or hard copy;

4. the policy had to be written in English;

5. the policy had to be available for review by project investigators no later than July 31, 2003.

#### Identifying Policy

Our primary source of alcohol and gambling policies was each school's student handbook. (For the purpose of this study, "student handbook" refers to the institution's primary informational document made available to current and prospective students.) The student handbook is a centralized forum for regulatory information and is a primary source of official school policies for students and parents, as well as the public. In addition, the concept of a student handbook is widespread, making handbooks a common information source across many schools. Many institutions distribute student handbooks to all incoming freshmen; therefore, most students are familiar and comfortable with accessing the handbook. Student handbooks also are widely available to the public.

When available, we used electronic versions (i.e., pdf or html) of each school's handbook; otherwise, we used a hard copy. Some schools, particularly large universities with many departments and/or divisions, did not have a single handbook that they distributed to all students. In these cases, we retrieved the school's policies from other official documents (e.g., code of conduct, policy manual, judicial procedures manual). Many schools also posted policy information (i.e., separate from the handbook) on their Web sites; we analyzed this information as a secondary source. We conducted an exhaustive search of each school's Web site using each site's integrated search engine and used keywords such as "alcohol," "drinking," "alcohol policy," "gambling," "wagering," "betting," "gambling policy," "substance use policy," "college (university) regulations," and "college (university) policies" to identify relevant sections of each Web site. Several sites did not include a search function; in such cases, we conducted a comprehensive visual search of the site. We also examined supplemental materials provided by schools (e.g., policy manuals, brochures, pamphlets, etc.) for comparison against handbooks and Web-based materials. We conducted a visual search of all hard-copy handbooks and supplemental policy materials and extracted all relevant information from these sources.

We systematically archived all of the Web-based and other electronic regulatory sources (e.g., pdf- and text-based student handbooks and policy manuals, html pages, etc.) from each school on a computer. We filed hard copy materials, such as student handbooks and policy manuals, by school and kept these documents on site.

### Policy Coding Procedure and Instrument

Investigators developed a coding instrument by studying alcohol and gambling policies from a variety of U.S. schools outside the current sample and identifying the underlying characteristics of the policies. These characteristics were reduced to 40 items that reflected the scope and focus of school alcohol and gambling policies. The items were converted into a coding instrument that included 25 variables for alcohol policy and 15 variables for gambling policy. This instrument included items to measure the presence of specific policies and establish whether the policies were punitive or rehabilitative. All variables used a nominal scale that included common characteristics of each school policy; response choices varied slightly with the focus of each variable. All of the variables were arranged on a six-page coding form.

To simplify coding and allow for within-school comparisons between different formats of policy dissemination (e.g. school handbook vs. school Web site), we separated each school's policy materials into three categories: (1) student handbooks (electronic or paper); (2) Web-based materials; and (3) supplementary materials (paper); a potential 357 documents required coding (three coding categories for each of 119 schools in sample = 357 potential documents). However, because not every school had documents available in all three coding categories, the final document count was 164. Specifically, at the end of our data collection process, we had collected 73 student handbooks, 70 Web-based policies, and 21 supplementary documents.

We assigned 11 coders the job of evaluating each school's alcohol and gambling policies. Each coder read a selection of policies and extracted relevant information in accordance with the coding form. The coding process proceeded as follows:

1. Each policy document was assigned to two of eleven eligible DOA coders randomly. Each assigned coder independently abstracted information from each assigned policy document and recorded this information on separate coding forms.

2. For each document, one member of the research team, designated as the "arbiter," compared the two coding forms and marked discrepant items.

3. The arbiter returned the marked coding forms to their respective coders and requested that coders reconsider their answers to the items in question. Upon reconsideration, coders were free to change their answers or keep their original answers.

4. Coders resubmitted their recoded documents to the arbiter who compared the discrepant items again. Discrepancies that remained were noted and resolved by the arbiter.

5. Once all discrepancies had been resolved, the policy assessments on the coding forms were entered into an SPSS database using a procedure that screened entries for out-of-range values and discrepancies in branching among items.

6. We assessed data entry reliability by selecting 10% of the cases in our database and rechecking each data entry point. Of the 680 items entered in these 17 randomly selected cases, there were no observed data entry errors.

Shaffer and his associates have used a similar process of information extraction, coding and arbitration successfully in other published studies [1,32].

## Results

Our analysis of college alcohol and gambling policies generated several types of results. First, we describe the results of our coding procedure, the final sample of schools and available policy information. Next, we examine the policy evidence across information sources by analyzing the consistency between the information provided by handbooks and Web materials. We then present the prevalence of individual policy items and the results of a factor analysis that explored the underlying dimensions of the policy variables. Finally, we analyze the relationships between policies and student drinking and gambling rates using information collected in the most recent Harvard School of Public Health College Alcohol Study (CAS) [6].

### Inter-Coder Concordance

We assessed inter-coder reliability by comparing the total number of discrepant coded items to the total number of coded items. As described previously, each policy was assigned to two of eleven eligible DOA coders randomly. The participation of eleven coders yielded 55 possible coding-pair combinations; each of these pairings coded at least one policy. Specifically, the number of policies coded by each coder-pair ranged from a minimum of one (n = 6) to a maximum of six (n = 3). Coders had up to two opportunities to code each document: (a) an initial round of coding; and (b) a second round of coding to reconsider any discrepant items identified by the arbiter after the initial round of coding. The arbiter made the final coding decision on 345 out of a total of 4,100 possible items. The coding process yielded a study-wide inter-coder reliability rate of 91.6%.

### College Sample

After thirty days had passed from our initial e-mail request, 46 of 119 schools had responded by sending hard copy materials. Eighteen of these 46 colleges sent materials completely unrelated to our request for school alcohol policies (e.g., applications for admission, school newsletters). Fourteen schools sent student handbooks, and another 14 schools sent other alcohol and/or gambling related (i.e., non-handbook) materials. Seventy-three schools did not respond to our request within thirty days. Subsequent to our follow-up telephone requests, we received student handbooks and supplemental materials from an additional 22 schools. This recruitment procedure resulted in 50 schools actively providing policy information for this study; for the remainder, policy information was obtained through other investigative procedures as described earlier (e.g., Web sites).

### Policy Sample

This study sought information on alcohol and gambling policy from a representative sample of 119 colleges across the U.S. We utilized three distinct common sources of information on school alcohol policy: student handbooks, school Web sites (non-handbook related) and supplementary materials (e.g., policy manuals, pamphlets). We collected a total of 164 policy-related documents from three sources: 73 policy documents from handbooks, 70 documents from school Web sites, and 21 from supplementary materials. Table 1 presents the sources of alcohol policy information for the schools in our sample. Forty schools presented their full alcohol policy in their handbook, 31 on their Web site, 2 in supplementary materials, and 44 through a combination of handbook, Web site and supplements. We were unable to locate any policy information for two schools in our sample; these schools did not respond to our requests for information.

**Table 1:** Sources of school alcohol policy information

**Number of Schools**	**Handbook Policies**	**Web site Policies**	**Supplemental Materials**	**No materials**
40				
31				
2				
25				
5				
11				
3				
2				
Total = 119	Total = 73	70	21	2

### Policy across Information Sources

We aggregated and analyzed policy information across sources because a preliminary examination revealed content differences among handbooks, Web sites [33] and supplementary materials. Aggregating information across sources provides the most extensive view of each college's policy strategy because it considers all modes of policy distribution. This strategy yields the most comprehensive policy search and identifies more policy mentions than is possible by examining only one policy source. To implement this strategy, we first constructed a new database that included data for schools with a handbook, a Web site or both (n = 115). Next we created a single record of policy mentions for the 28 schools with both handbooks and Web materials by aggregating policies across sources. This database assimilated the unique handbook and Web variables into a single set of "recompiled" variables, reflecting the total number of policies attributable to either the handbook or the Web. To compare the "added value" of school Web sites (i.e., policy information presented on the Web that was not presented in the handbook), we summed the policies reflected by the recompiled variables and then subtracted the policies contained in the handbook-only variables. Of 263 total policy items present, we collected 198 (75%) policy items from student handbooks and 65 (25%) additional policy items from school Web sites that were not available in handbooks.

To determine the added contribution of supplemental materials (i.e., policy information presented in the supplements that was unavailable elsewhere), we created another set of recompiled variables following the previously outlined procedure. These variables reflected the total number of policies identified for the three schools with all three types of sources (i.e., handbooks, Web materials and supplements). We summed the policies reflected by the recompiled variables and then subtracted the policies contributed by handbooks and Web sources; this procedure revealed that supplemental materials contributed 4 of 30 (13.3%) policy items.

Although school Web sites provide a substantial amount of alcohol policy information that is not contained in the primary document customarily provided to students (i.e., handbook), the overall added contribution of the school Web site in presenting policy information varied among schools. For example, one school's Web site contained an additional eight alcohol regulations that were not included in the handbook; however, several schools' sites contained no additional information. In addition, the type of information that was presented only on Web sites also varied: while most information pertained mainly to secondary alcohol policies (e.g., school-sponsored events and drinking regulations for drinking-aged students), some schools chose to present vital alcohol policy information (e.g., stating that all drinking is prohibited for students <21) on their Web site only (n = 2). Thus, although handbooks and Web sites are both important sources of alcohol policy information and supplements contribute little additional information, consistency across sources varies. The following analyses assess the agreement of information found in multiple sources.

### Handbook-Web Concordance

As mentioned earlier, of the 117 colleges for which we had information, all 117 (100%) had a written policy on student alcohol consumption and 26 schools (22%) had a student gambling policy. Because all schools had a written alcohol policy (and relatively few schools had a gambling policy), the following analyses focus on alcohol policies.

Determining concordance between handbook and Web sources is important because administrators might be unaware of inconsistencies between official school documents. In addition, contradictory information can mislead students and potential applicants. We assessed the concordance between sources of college alcohol policy materials by determining the level of agreement (i.e., presence or absence of policy information) between handbooks and Web materials; that is, we compared the content of each type of document to identify differences in the presentation of each school's policy information between sources. We did not extend this particular analysis to include supplemental materials because, as we noted before, only a small number of schools (n = 3) had all three types of sources.

Twenty-eight schools had both a handbook and Web materials; for each of these 28 schools we determined the absence or presence of the 25 alcohol policy variables in each source. We predetermined that a concordance rate of 85% would indicate a high level of agreement between documents. To be considered in agreement, complementary information had to be found in (or absent from) both sources; in cases where this requirement was not satisfied, the policies were considered in disagreement. Using these criteria, we determined that three policy variables (i.e., 12% of the policy variables) were mentioned often and were present in both handbooks and on Web sites, and consequently, showed high agreement. Either type of information resource seldom mentioned ten policy variables (i.e., 40% of the policy variables), therefore, also exhibiting high agreement. The remaining 12 policy variables (i.e., 48% of the policy variables) were often mentioned, but not consistently by both sources, indicating low agreement.

Table 2 presents the three "high agreement" alcohol policies that were mentioned consistently in both handbooks and Web materials. Variables that fell into this category generally measured broad school policies (i.e., the existence of an alcohol policy). As Table 2 illustrates, schools consistently made these types of alcohol policies available to the public in both print and electronic form, making this information highly accessible.

**Table 2:** "High Agreement" Alcohol Policies, Often Mentioned in Both School Handbooks and Web Materials (N = 28)

**Policy**	**% of schools, HB only**	**% of schools, Web site only**	**% of schools, HB and Web site**	**% of schools, no mention in HB or Web site**
Is there an alcohol policy?	0	0	100.0	0
Alcohol is prohibited on campus for students <21	7.1	7.1	85.7	0
Alcohol is allowed at sanctioned events for students ≥ 21	0	0	87.5	12.5

Policy variables that were rarely mentioned in handbooks and Web materials appear in Table 3. These variables primarily measured on- and off- campus alcohol consumption restrictions and school recovery polices regarding student alcohol use. These policies are in "high agreement," because they were seldom mentioned: as Table 3 demonstrates, this information was missing from handbooks *and *Web sites in nearly all cases.

**Table 3:** "High Agreement" Policy Variables, Rarely Mentioned in School Handbooks and Web Materials (N = 28)

**Policy**	**% of schools, HB only**	**% of schools, Web site only**	**% of schools, HB and Web site**	**% of schools, no mention in HB or Web site**
Alcohol is prohibited off-campus for students ≥ 21	0	0	0	100.0
Alcohol quantity limits at off-campus events	0	0	3.6	96.4
Policy on container restrictions at off-campus events	3.6	0	0	96.4
Policy on leave of absence for recovery	3.6	0	0	96.4
Policy allowing students to participate in recovery while living in dorm	3.6	3.6	0	92.9
Attendance restrictions for hosted events	0	10.7	3.6	85.7
Policy on students with an alcohol problem upon entering school	7.1	3.6	0	89.3
Policy on students who develop an alcohol problem while in school	10.7	3.6	0	85.7
Policy on students already in recovery upon entry to school	10.7	3.6	0	85.7
Policy on students who enter recovery while in school	10.7	3.6	0	85.7

Table 4 presents variables that were mentioned occasionally (i.e., concordance <85%) in handbooks or Web materials. The policies in this category primarily address consumption and event restrictions and student recovery. Table 4 illustrates that we observed considerable inconsistencies in schools' methods of distribution of these types of policies.

**Table 4:** "Low Agreement" Policy Variables, Mentioned Inconsistently in School Handbooks and Web Materials (N = 28)

**Policy**	**% of schools, HB only**	**% of schools, Web site only**	**% of schools, HB and Web site**	**% of schools, no mention in HB or Web site**
Alcohol is prohibited on-campus for students ≥ 21	0	7.1	10.7	82.1
Off-campus alcohol restrictions in place for students ≥ 21	10.7	7.1	3.6	78.6
School policy is to defer to local laws on alcohol consumption	14.3	10.7	3.6	71.4
Policy on alcohol quantity limits at events	17.9	10.7	3.6	67.9
Attendance restrictions for school sanctioned events	21.4	10.7	3.6	64.3
Campus operates an alcohol recovery program	32.1	7.1	3.6	57.1
Policy on alcohol-free campus housing	25.0	25.0	3.6	46.4
Document makes clear other ways by which the school makes students aware of the official alcohol policy	25.0	17.9	21.4	35.7
Policy on container restrictions on campus	21.4	21.4	21.4	35.7
Campus makes referrals to off-campus recovery programs	25.0	21.4	28.6	25.0
On-campus alcohol restrictions in place for students ≥ 21	13.0	26.1	56.5	4.3
Policy on alcohol at on-campus sanctioned events for students ≥ 21	17.9	21.4	57.1	3.6

### Identifying the Underlying Dimensions of College Policy

As noted earlier, the coding process revealed that all 117 colleges (i.e., 100% of the schools for which information was available) in this sample had a written policy on student alcohol consumption, but only 26 (22%) had a published policy that addressed gambling. The small number of schools with gambling policies precludes confident analysis of the dimensional composition of our gambling variables; therefore, we applied the factor analysis that follows only to alcohol policy variables.

Three policy variables represented a multi-dimensional measurement strategy to yield detailed policy information. Consequently, we collapsed these three redundant policy items into the primary or gate items from which they originated (e.g., "alcohol is prohibited on-campus for students ≥ 21" and "on-campus alcohol restrictions in place for students ≥ 21" became "policy on alcohol use on-campus for students ≥ 21). This resulted in 22 alcohol policy variables in all remaining analyses. These dependent variables all measured different aspects of school alcohol policies (e.g., policy presence, content, and target). To empirically examine the underlying dimensions reflected by our variables, we conducted an exploratory factor analysis. This procedure employed an initial factor extraction (i.e., component matrix) and then an orthogonal rotation to simple structure. We selected the Varimax rotation to maximize the variance of loadings within factors and minimize the covariance across factors. The orthogonal solution identified eight policy clusters with Eigenvalues greater than 1.0 that explained 72.36% of the total variation. This explained variance lies within the 50–75% useful range suggested by Overall and Klett (1972)[34]. Consequently, we concluded that our factor analysis provided a valid identification of the policy clusters that underlie college alcohol and gambling regulations.

Table 5 presents the structure of the interrelationships among policies. To facilitate interpretation, the table reports only factor loadings ≥ 0.50 (i.e., policies with loadings in this range correlate .50 or greater with a composite measure of the overall dimension).

**Table 5:** Orthogonal Factor Structure and Items Loading ≥ 0.50 on Each Factor

***Variable***	**Factor Loading**	**% Variance Explained**
**Factor 1 – School policy and the law**		4.83
Does the school alcohol policy defer in full to local law?	.88	
**Factor 2 – Prohibition policies**		5.43
Does the policy state that alcohol is prohibited for students <21?	.85	
Does the policy state that alcohol is restricted on-campus for students ≥ 21?	.76	
**Factor 3 – Policies for legal-aged drinkers**		6.65
Is alcohol is allowed at sanctioned on-campus events for students ≥ 21?	.75	
Is alcohol prohibited off-campus for students ≥ 21?	.74	
**Factor 4 – Limits and restrictions – on-campus**		9.13
Does the policy state whether the school offers alcohol-free campus housing?	.77	
Does the policy address alcohol container restrictions on campus?	.74	
Does the policy address alcohol quantity limits (i.e., total alcohol available) at on-campus sanctioned events?	.57	
**Factor 5 – Events policies**		7.20
Are there attendance restrictions for off-campus sanctioned events?	.81	
Are there attendance restrictions for on-campus sanctioned events?	.78	
Are there restrictions on off-campus alcohol use for students ≥ 21?	.56	
**Factor 6 – Limits and restrictions – off-campus**		13.50
Is there policy on alcohol quantity limits (i.e., total alcohol available) at off-campus sanctioned events?	.81	
Is there policy on alcohol container restrictions off-campus?	.79	
Is there policy that permits students a leave of absence to participate in a recovery program?	.50	
**Factor 7 – Recovery recognition policies**		20.59
Is there policy on students who enter alcohol recovery while attending?	.90	
Is there policy on students who are in alcohol recovery upon entry?	.88	
Is there policy on students who have an alcohol problem upon entry?	.87	
Is there policy on students who develop an alcohol problem after entry?	.79	
Is there policy that permits students in an alcohol recovery program to live in a dormitory on campus?	.73	
**Factor 8 – Recovery facilitation**		5.03
Does the campus makes referrals to an off-campus recovery program for students with alcohol use disorders?	.74	
Does the campus operate a recovery program for students with alcohol use disorders?	.73	

The eight factors are ordered according to the number of policies measuring the overall domain; that is, factors containing general, or "blanket," policy items are listed first, followed by factors containing more specific policy items. Factor 1 (i.e., *School policy and the law*) contained only one item that loaded ≥ 0.50 and explained 4.83% of the total variance. Many schools deferred to local law entirely and did not publish other policies that were unique to the school. Factor 1 identifies this deference policy as a unique dimension. Factor 2 (i.e., *Prohibition policies*) expands on deference to local law and presents additional school policies that prohibit alcohol for underage and legal age students. These items accounted for 5.43% of the total variance. Factor 3 includes policies that extend restrictions to include drinking by students of legal age (i.e., *Policies for legal-aged drinkers*). Items in Factor 3 explained 6.65% of the total variance. Factors 1, 2 and 3 include alcohol policies that focus on the legal status (i.e., legal age) of students; in addition, for those of legal age, these policies range from no school specific policies to prohibitions for students who are old enough to drink legally.

Factors 4 through 6 include policy variables directed to alcohol use on-campus and off-campus. Factor 4 (i.e., *Limits and restrictions – on campus*) provides specific guidance about where on-campus students can drink and how much alcohol is available (i.e., housing and container and quantity restrictions); these items accounted for 9.13% of the total variance. Factor 5 policies (i.e., *Events policies*) accounted for 7.20% of the total variance and focus primarily on restrictions for on- and off-campus events. Factor 6 (i.e., *Limits and restrictions – off *campus) accounted for 13.50% of the total variance and includes policies that regulate off-campus activities (i.e., alcohol quantities, containers and leaves of absence).

Different from the first 6 factors, factors 7 and 8 focus on student recovery. Factor 7 policies (i.e., *Recovery recognition policies*) recognize that students can have alcohol related problems that require recovery, and that these problems can exist before entering college or develop during college; these items accounted for 20.59% of the total variance in the data. Finally, Factor 8 (i.e., *Recovery facilitation*) accounted for 5.03% of the total variance and includes policies that reflect how the school participates in the recovery process (i.e., triage or treatment). One item (i.e., "how does the campus inform students of the official school policy") failed to load ≥ 0.50 on any factor and was excluded from the final analysis.

### Alcohol Policy Prevalence

College alcohol policies varied widely. Table 6 summarizes the prevalence of alcohol-related policy and the mean prevalence of alcohol policies within each factor. The prevalence of alcohol policies ranged from 100% (i.e., the presence of an alcohol use policy) to 1.7% (i.e., policy that permits a leave of absence to participate in a recovery program). The mean prevalence for the eight policy factors ranged from 92.3% (i.e., *Prohibition policies*) to 5.4% (i.e., *Limits and restrictions*-*off-campus*).

**Table 6:** Prevalence of College Alcohol Policies and Policy Attributes

**Policy & Policy Attributes**	**Prevalence % (N)**
School has a written policy on alcohol use	100 (117)
Policy states that alcohol is prohibited for students <21	97.4 (114)
Policy on alcohol use on-campus for students ≥ 21	87.2 (102)
Policy addresses alcohol at sanctioned on-campus events for students ≥ 21	68.4 (80)
Campus makes referrals to an off-campus recovery program for students with alcohol use disorders^♠^	57.3 (67)
Policy addresses alcohol container restrictions on-campus	50.4 (59)
Policy makes clear how the campus informs students of the official school alcohol policy	43.6 (51)
Campus operates a recovery program for students with alcohol use disorders^♠^	28.2 (33)
School alcohol policy defers in full to local law	26.5 (31)
Policy addresses alcohol quantity limits (i.e., total alcohol available) at on-campus sanctioned events	26.5 (31)
School offers alcohol-free campus housing	22.2 (26)
Restrictions on off-campus alcohol use for students ≥ 21	21.4 (25)
Attendance restrictions for on-campus sanctioned events	20.5 (24)
Policy on students who develop an alcohol problem after entry^♠^	10.3 (12)
Policy on alcohol quantity limits (i.e., total alcohol available) at off-campus sanctioned events	8.5 (10)
Policy on students who enter alcohol recovery while attending^♠^	7.7 (9)
Attendance restrictions for off-campus sanctioned events	7.7 (9)
Policy on students who are in alcohol recovery upon entry^♠^	6.0 (7)
Policy on alcohol container restrictions off-campus	6.0 (7)
Policy permits students in an alcohol recovery program to live in a dormitory on campus^♠^	5.1 (6)
Policy on students who have an alcohol problem upon entry^♠^	3.4 (4)
Alcohol is prohibited off-campus for students ≥ 21	3.4 (4)
Policy permits students a leave of absence to participate in a recovery program^♠^	1.7 (2)

**Policy Factor**	**Mean Prevalence (%)**

Factor 2 – Prohibition policies	92.3
Factor 8 – Recovery facilitation^♦^	42.8
Factor 3 – Policies for legal-aged drinkers	35.9
Factor 4 – Limits and restrictions – on-campus	33.0
Factor 1 – School policy and the law	26.5
Factor 5 – Events policies	16.5
Factor 7 – Recovery recognition policies^♦^	6.5
Factor 6 – Limits and restrictions – off-campus	5.4

^♠^Recovery-oriented policy; ^♦ ^Recovery-oriented factor

### Policy, Binge Drinking and Gambling

We conducted several analyses to determine the nature of relationships between student alcohol consumption, gambling behavior and policy content. As the factor analysis above illustrates and the relative prevalence of policies confirms, college alcohol-related policies are primarily intended to prevent, reduce or restrict alcohol use among students on college campuses. To test the relationships between alcohol policies and student drinking behaviour, we compared the mean binge drinking rates of students at schools with and without each policy variable. We obtained the mean binge drinking rates of the schools in our sample from the dataset used in Wechsler et al.'s Harvard School of Public Health College Alcohol Study (CAS)[6]. Because this is one of the first studies of college policies, we sought to identify as many potential relationships between policy, drinking and gambling as possible; therefore we set a liberal alpha level (α = .1) for this analysis. A one-way analysis of variance (ANOVA) revealed that four of the 22 policy variables had significant relationships with binge drinking rates at the colleges in our sample (see Table 7).

**Table 7:** Mean Binge Drinking Rates (%) and Alcohol Policy Variables

**Policy variable**	**Schools with no policy mention (N)**	**Schools with no policy restrictions (N)**	**Schools with restrictions policy (N)**	**Schools with prohibition policy (N)**	**F**	**df**
Policy on alcohol use on-campus for students ≥ 21	n/a (0)	39% (15)	47% (77)	36% (24)	7.07***	2,113
Policy on alcohol at on-campus sanctioned events for students ≥ 21	44% (37)	46% (64)	n/a (0)	33% (15)	5.25***	2,113
Policy on students already in recovery upon entry to school	43% (109)	56% (6)	57% (1)	n/a (0)	2.89*	2,113
	**No mention (N)**		**Alcohol free housing available (N)**			
Policy on alcohol-free campus housing	42% (90)		49% (26)		4.01**	1,114

* = *p *< .1; ** = *p *< .01; *** = *p *< .001

Schools that had either no policy restrictions or a prohibition policy for on-campus alcohol use by students ≥ 21 had lower mean past-month student binge drinking rates (39% and 36%, respectively) compared to schools that employed an intermediate level of restrictive policies (47%) (F = 7.07, df = 2,113, p < .001). Schools that allowed or did not mention alcohol use at on-campus sanctioned events for students ≥ 21 had a higher mean binge drinking rate (46% and 44%, respectively) than schools that prohibited legal drinking at events (33%) (F = 5.25, df = 2,113, p < .001). Schools that offered alcohol-free housing had a higher mean student binge drinking rate of 49% compared to schools that did not mention alcohol-free housing which had a binge rate of 42% (F = 4.01, df = 1,114, p < .01). Two other alcohol policy variables evidenced significant relationships with student binge drinking behaviors, but these policies lacked widespread implementation at a large number of schools. Schools that specifically allowed a leave of absence for a student to participate in recovery activities (n = 2) evidenced a higher mean binge drinking rate (69%) than schools that did not mention such a policy (43%, n = 114). Keeping the small number of schools in mind, it is worth noting that schools that prohibited off-campus alcohol consumption for students ≥ 21 (n = 4) had a lower mean binge rate (10%) than schools without this provision (45%, n = 112).

There were not enough schools with gambling policies to permit a detailed analysis of the relationship between policies and student gambling behavior; therefore instead of conducting an analysis of the relationship between gambling behavior and individual policy variables, we only were able to assess gambling behavior based on whether schools had a gambling policy. Using prevalence data from LaBrie et al.'s (2003) [31] recent study of student gambling behavior, we determined that no significant difference in mean past-year student gambling behavior existed between schools with a written policy on gambling (i.e., prohibitive or restrictive) and schools with no mention of gambling policy (i.e., approximately 40% regardless of policy presence).

### Unanticipated Policy Effects: Alcohol Policy can Influence Gambling Participation

Long ago, Pigou [35] noted that public policies can have unanticipated effects; policy intended to regulate one set of behaviors can influence other patterns of behavior. To test the relationships between alcohol policies and student gambling behavior, we compared the mean past-year gambling rate at schools with each alcohol policy to schools without the policy. As before, we used a liberal alpha (α = .1) to identify all potential relationships. A one-way analysis of variance (ANOVA) revealed that four of the 22 alcohol policy variables had significant relationships with the proportion of students who gambled in the past-year school year (see Table 8).

**Table 8:** Mean Past-Year Student Gambling Participation Rate (%) and Alcohol Policy Variables

**Policy variable**	**Schools with no policy mention (N)**	**Schools with no policy restrictions (N)**	**Schools with restrictions policy (N)**	**Schools with prohibition policy (N)**	**F**	**df**
Policy on alcohol use on-campus for students ≥ 21	n/a	46% (15)	40% (77)	41% (24)	2.64*	2,113
Policy on alcohol at on-campus sanctioned events for students ≥ 21	44% (37)	40% (64)	n/a (0)	35% (15)	5.17***	2,113
	**No mention (N)**		**Limits (N)**			
Policy on alcohol quantity limits at on-campus events	42% (85)		37% (31)		5.39***	1,114
Policy on off-campus alcohol restrictions for students ≥ 21	42% (91)		38% (25)		2.94*	1,114

* = *p *< .08; ** = *p *< .01; *** = *p *< .001

Schools that restricted or prohibited on-campus alcohol use for students over 21 evidenced similar mean past-year student gambling participation rates (i.e., 40% and 41%, respectively), and schools with no restrictive policy evidenced a higher student gambling participation rate (46%) (F = 2.64, df = 2,113, p < .08). Schools that did not mention or allowed alcohol at on-campus events for legal drinkers exhibited a higher mean gambling participation rate (i.e., 44% and 40%, respectively) than schools that prohibited alcohol at on-campus events (35%) (F = 5.17, df = 2,113, p < .001). Similarly, schools that did not limit the quantity of alcohol available at events showed higher past-year gambling participation among students (42%) compared to schools with no such provision (37%) (F = 5.39, df = 1,114, p < .001). Schools that had off-campus alcohol restrictions for legal-aged drinkers had a gambling rate of 38% while schools that did not had a mean gambling rate of 42% (F = 2.94, df = 1,114, p < .1).

Two other alcohol policy variables evidenced significant relationships with student gambling behaviors, but these policies were not widely implemented throughout the sample. Schools that did not expressly prohibit alcohol consumption for underage drinkers (n = 3) evidenced a mean past-year student gambling rate of 51%, while schools that prohibited underage drinking (n = 116) had a gambling rate of 41% (F = 3.19, df = 1,114, p < .1). Schools that banned all alcohol consumption, whether on- or off-campus (n = 4) evidenced a lower gambling rate (30%) than schools that allowed at least some drinking (41%, n = 112) (F = 5.80, df = 1,114, p < .05).

In addition to the direct relationships between policy variables and binge drinking and gambling, three alcohol policy variables evidenced unexpected interaction or intensification effects when gambling policies also were present. Schools that had both a policy prohibiting or restricting gambling activity among students *and *a policy prohibiting on-campus legal-aged drinking (n = 9) had a mean binge drinking rate of 29% – much lower than schools with just an alcohol policy (40%, n = 15), a gambling policy (47%, n = 17) or neither (45%, n = 75) (F = 3.15, df = 1,112, p < .1). Schools with both a gambling policy *and *a policy prohibiting alcohol at on-campus events (n = 6) had a significantly lower mean binge rate (22%) than schools with just an alcohol policy (40%, n = 9), a gambling policy (46%, n = 20) or neither (45%, n = 81) (F = 5.88, df = 1,112, p < .05). Schools that prohibited alcohol at on-campus events also evidenced significantly lower past-year student gambling rates than schools without such prohibitions: 30% versus 38% or higher (F = 8.63, df = 1,112, p < .05).

## Discussion

Using written (i.e., hard copy) and Web based sources, this study examined the nature and extent of alcohol and gambling-related policies among a representative sample of U.S. colleges. Every school in this representative sample had at least one alcohol use policy; however, few schools (i.e., 26 of 117; 22%) had at least one gambling policy available. The relative rarity of gambling-related policies on college campuses represents a lost opportunity by school administrators to (a) prevent or limit disordered gambling among students and (b) facilitate recovery for students in need of gambling treatment. A recent study showed that, while not as prevalent as previously thought, gambling on college campuses is still quite common, with 42% of students having gambled in the past year and 2.6% gambling weekly or more [31]. The frequency with which gambling occurs on college campuses could be indicative of lingering misconceptions about gambling outcomes among student populations. For example, research has shown that gamblers are largely unaware of the probabilities associated with various forms of gambling (e.g., Ladouceur et. al. 1996[36]; Rogers 1998[37]); this circumstance leaves gamblers susceptible to cognitive errors [38]; [39]; [40] and suggests that gambling behavior is largely driven by social factors and injunctive norms (i.e., the tendency to engage in gambling as a function of personal perceptions of society's acceptance of gambling) [41,42,11]. By failing to implement comprehensive restrictive and recovery-based gambling policies and neglecting to educate students about the probabilities associated with gambling as well as the dangers of excessive gambling, school administrators are overlooking an important and potentially destructive problem that faces many of today's students. Future policy-based education and recovery initiatives might be able to effectively reduce student gambling behaviors; however, given the current dearth of gambling policies, we cannot determine whether school policies can effectively reduce at-risk gambling behaviors on college campuses.

All the schools in our sample recognized the need for some type of alcohol policy; however, the presence of more targeted policies varied considerably. Although this variation might reflect different policymaking strategies across institutions, it also could result from a variety of other influences, including the lack of federal standards guiding the creation of alcohol policy on college campuses. The absence of policy guidelines leaves administrators with a wide range of options about how to best address student substance use and abuse. Some administrators might prefer to match policy to their perception of local needs, while others might welcome policy guidelines. Both of these circumstances encourage additional research designed to help guide administrators to identify effective policies (e.g., "best practices").

Alcohol policies ranged from comprehensive restrictions and prohibition to liberal acceptance of student alcohol use. Policies encouraging recovery among students with alcohol use disorders were decidedly absent from our sample. For example, only 57.3% of schools expressed in writing that they made referrals to alcohol recovery services; all other recovery-oriented policy provisions were in place at fewer than 30% of schools, with two-thirds of these policies in effect at fewer than 10% of schools. Examination of the mean prevalence of the eight policy factors provides additional support for this finding. The mean prevalence of recovery recognition policies was 6.5% (i.e., factor 7). Although recovery facilitation policies were more common, with a mean prevalence of 42.8% (i.e., factor 8), this rate simply reflects that many schools report making referrals to outside treatment facilities. In contrast, the prevalence of prohibition policies was 92.3% (i.e., factor 1). This limited of consideration for student recovery and emphasis on punitive and prohibitive measures might reflect an underlying institutional bias against accommodating students with special needs and an unsupportive atmosphere for those who are at most risk for developing alcohol problems. The higher prevalence of policies that direct referrals to outside treatment resources might indicate an eagerness among administrators to export students with addiction problems to non-school affiliated assistance. Alternatively, placing little emphasis on recovery might simply represent a lack of understanding about addictive behaviors and addiction recovery among school administrators.

Even though the results of this study indicate that schools with recovery policies can evidence higher rates of binge drinking, this rate might reflect a pre-existing campus problem (i.e., policy as a consequence of behavior) rather than be a result of recovery policy implementation. Future policy research needs to examine whether a better balance between punishment and treatment policies will yield improved student health services, less substance misuse and, consequently, a better campus life for all students.

As hypothesized, there were considerable and important differences between the information that was available in handbooks and on the Web. This observation is not surprising because there are few requirements guiding the creation of school substance use and gambling policies and no standards requiring consistency among sources. Also, the factor analysis illustrated that alcohol policies currently concentrate primarily upon prevention and punishment, and devote relatively limited attention to student recovery. Finally, as we expected, schools with no policy restrictions on alcohol consumption or restrictive alcohol policies often experienced higher levels of binge drinking among students than schools with prohibitive alcohol policies. Nevertheless, alcohol policies were associated inconsistently with student binge drinking rates; few policy variables exerted influence on patterns of student drinking. In some cases, policies designed to reduce student alcohol consumption showed an opposite effect.

### Absence of Shared Standards and Model Policies

The results of this policy analysis suggest diverse and perhaps ambivalent tolerance toward alcohol use among U.S. colleges that has led to a deep underlying problem: there is a lack of model policy guidelines to assist colleges in (a) preventing addictive behaviors among students and (b) providing assistance to students already struggling with addiction. Optimally, if available, such guidelines would provide school administrators with "best practice" model policies that address the many aspects of addiction among students (e.g., permitted and prohibited substance use on campus, legal matters, parental notification, treatment options, financial issues, academic issues, etc.) and would outline strategies for implementing and enforcing such policies. In the absence of an explicit regulatory framework (i.e., the current policy environment), schools are left to regulate addictive behaviors based upon the local attitudes and expectations of communities and implicit moral values held by school administrators. This circumstance can lead colleges to install policies intended to have immediate and drastic results (i.e., prevention and punishment policies) while neglecting policies that would promote recovery and provide lasting benefit to students. The absence of evidence-based policy leaves school administrators in a position of promulgating policy that might have effects that are contrary to their intentions. For example, the Boston Globe reported on October 30, 2002 that, "Forty-seven Massachusetts colleges will sign onto a statewide campaign today designed to punish students who abuse alcohol and to cut down on the rate of binge drinking on campuses by providing alcohol-awareness training for students, athletes, and Greek system members"[43]. This type of policy imposes blind restrictions and punitive measures on students without considering the underlying factors motivating student drinking (e.g., campus environment) or the need for specialized programs (e.g., recovery programs) to assist in reducing student drinking. Thus, even when colleges have existing policies that address alcohol, drug use or gambling, it is likely that the number of policies related to students involved with or seeking recovery programs is much more limited. It is interesting to consider that colleges routinely provide remedial courses in expository writing to help students cope with the demands of academics but resort to exclusion or punishment for students who fail to adequately cope with an academic environment where 44% of students binge drink [6].

### Inconsistent Policy Presentation

The prevalence of alcohol-related problems at U.S. colleges highlights the need for comprehensive student policies addressing all aspects of alcohol use. Further, for policy to be effective students need to be aware of and understand school requirements; this requires that students know how to access policy information. Because schools currently are not required to disseminate alcohol policies in any particular form or through any specific medium, the availability of existing policy information can vary substantially among schools. Though most commonly printed in student handbooks, substance use policies also appear in policy manuals, pamphlets and, increasingly, on school Web sites. One recent study of college Web-based alcohol policy information [33] found that 50 of 52 schools included alcohol policy information on their Web site; unfortunately, schools rarely consolidated this information onto a single Web page for easy viewing. The result was that alcohol policy information was often incomplete and/or difficult to access [33]. Although the Internet provides an excellent opportunity for schools to reach technology-savvy students and parents, individuals seeking policy information on the Web are likely to face disorganization and user-unfriendly designs. Thus, while students and parents might expect to access alcohol or gambling policies by turning to a college's student handbook or Web site, the results of this study suggest that their success in locating the desired information will vary considerably. Adoption of generally accepted standards for policy dissemination could increase student awareness, and consequently, compliance with college rules.

Tables 2 through 4 illustrate that schools present different types of alcohol policy information in their handbooks and Web sites; sometimes this information agrees across sources, and often it does not. For some policy variables, this discrepancy is problematic. For example, most schools (82.1%) did not report on the presence or absence of alcohol-free campus housing at all. It is difficult to assess whether an absence of policy indicates a tacit acceptance of an activity (i.e., legal-aged alcohol consumption). More likely, however, it indicates that at these schools some drinking is permissible among students over age 21. For other policies, schools' lack of consistency in reporting uniform policy creates different problems. For example, as Table 4 illustrates, handbooks and Web sites mislead people inquiring about on-campus alcohol-free housing 25–50% of the time, at schools that have such a policy, depending upon the information source (i.e., handbook or Web site). Many schools (46.4%) did not mention alcohol-free housing at all; it is unclear whether these schools have alcohol-free on-campus housing, or simply lack an explicit policy addressing the matter.

This study reveals that to gain a complete understanding of the components of a school's alcohol policy, it is necessary to consult both the handbook and the school Web site whenever possible. However, schools do not alert students and parents to this fact, and it is unreasonable for school administrators to expect inquiring persons to conduct complex searches for information that is considered to be freely available in the public domain.

### Policy Content: Prohibition, Punishment and Recovery

The results of the factor analysis provide a stark portrait of the current composition of college policies on potentially addictive behaviors. This analysis reveals that six of eight factors contained prohibitive and/or punitive policy variables. Although the factor analysis merely revealed the underlying psychometric properties of our instrument, the prevalence of specific policy variables across schools provides additional support for this finding. Whereas the results suggest that prohibition-oriented policies have been effective in reducing binge drinking and gambling under certain conditions, the relative scarcity of recovery-oriented policies prevents us from properly comparing the efficacy of these two strategies.

### Policy, Binge Drinking and Gambling

The analysis of policy variables and binge drinking rates revealed a variety of relationships among policy variables and student drinking behaviors. Specifically, four policy variables related to student binge drinking (see Table 7), and four alcohol policy variables related to student gambling behaviors (see Table 8). Interpretation of some of these relationships is relatively straightforward. For example, schools that prohibited alcohol at on-campus events experienced less binge drinking than other schools. This finding suggests that most students will abide by school policy. However, other relationships are more difficult to interpret: schools that restricted legal-age drinking had higher binge drinking rates than schools that did not make restrictions for students ≥ 21. Perhaps, when forbidden, students find alcohol to be more desirable. Alternatively, students of legal age might feel constrained by prohibitions and, as a result, drink more often to excess than they would if the opportunity to drink was commonplace. This pattern of drinking was commonly observed during the Volstead Act (i.e., national prohibition from 1920–1933). Many people did not drink because it was illegal, but those who did tended to drink to excess [44]. Just as with the Volstead Act, determining the real effect of school alcohol policies is difficult because many other factors (e.g., how long the policy has been in effect, state or local culture, etc.) influence drinking. The relationship between policy and drinking can be counterintuitive. For example, schools that offered alcohol-free campus housing evidenced significantly higher mean student binge drinking than schools that did not mention alcohol-free housing (49% vs. 42%, respectively). Observers might expect schools promoting alcohol-free dormitories to evidence lower binge drinking; alternatively, schools with "dry" housing might better recognize alcohol related problems on their campus and set policy intended to counter these problems among their students. Accordingly, the 49% binge rate observed in this study could reflect a significant improvement in the rate of binge drinking for these schools. Without longitudinal data, however, this analysis is beyond the scope of this study. Four schools in the sample indicated that they prohibited all alcohol use by students both on- and off-campus. These schools had a significantly lower mean binge drinking rate than other schools (10% vs. 45% respectively), suggesting that prohibition discouraged drinking among the majority of the student body; alternatively, these schools might attract students less interested in drinking. Three of the schools were religiously affiliated, and the fourth admitted primarily African-American students; both minority status and religiosity are cultural factors that have been shown to be associated with decreased substance abuse [45,46].

### Unanticipated Policy Effects

The interaction effects observed among alcohol policy variables and the presence of gambling policy on binge drinking behavior and past-year student gambling behavior presents an interesting and unanticipated finding. Because schools that have prohibitive alcohol policies *and *prohibitive gambling policies evidence lower mean rates of binge drinking among students than other schools, restrictive policies seem to have the intended effect of countering potentially destructive behaviors among students. However, other conditions such as cultural factors also play an important role in determining student behaviors. For example, students that choose to attend schools with rigorous policy provisions might be intrinsically more likely to refrain from excessive alcohol consumption for ethical or religious reasons. Further, five of the six schools in our sample that had both (a) a policy prohibiting alcohol at on-campus events and (b) a prohibitive gambling policy also had small enrollment (i.e., below the 50^th ^percentile among schools in our sample); four of these six schools were state-operated. Underlying characteristics of students who seek out small state schools might be associated with the reduced binge rates reported among these institutions. These results suggest that, despite the role for shared policy guidelines, schools will benefit from analyzing the composition of their student body and tailoring new and modified alcohol policies to students' specific characteristics. Although competing explanations prevent the establishment of concrete cause-and-effect relationships in this study, the observed interaction effects between alcohol and gambling policies provide significant impetus for future research into effective policy strategy on college campuses.

### Implications

From an observer's perspective, it appears that the many policy inconsistencies – and policy presentation inconsistencies – observed in this study reflect reactive policymaking strategies that are not guided by empirical evidence. The evidence suggests that effective school alcohol and drug policies, and student awareness of these policies, are important for many reasons. For example, college-aged individuals in recovery are extremely vulnerable to relapse; in addition to the generally high rate of relapse during the first year of recovery [47,48], this circumstance exists in part because of their age and the prevalence of drug and alcohol abuse among their peers. Students need to be aware of the potential health and treatment options that are available to them on-campus if relapse occurs. Young people have not had alcohol use disorders as long as their adult counterparts because of their age; similarly, college students recovering from an alcohol use disorder have not been healing for very long. Relapse can generate harmful financial, academic and other consequences that can impart severe restrictions upon students' actions, both on and off campus. Consequently, it is valuable for students struggling with addiction to be able to access specific school policies before they enter a college or university; students who develop an alcohol problem after they are enrolled in college also need access to this information, as well as policies governing recovery-seeking. Although it is possible that policies can reduce alcohol abuse and dependence, comprehensive policies governing alcohol consumption among students hold the greatest potential to reduce pre-morbid and sub-clinical alcohol use on college campuses. Addiction models generally propose that while sub-clinical alcohol use and gambling can ultimately lead to a pathological state, pre-morbid subjects also can move away from pathology and maintain controlled behavior or abstinence [49-51]. This can occur through the influence of social setting attributes, including policy directives. Nevertheless, the high rate of drinking and binge drinking among college students has continued despite evidence that schools have devoted increased attention to promoting alcohol awareness and prevention recently (e.g., Wechsler et al. 2002 [6]). Various factors, such as a lack of agreement among school policies, unbalanced policies (e.g., policies that focus on punishment but not recovery), or a failure on the part of school administrators to enforce stated rules could undermine a cohesive alcohol policy and contribute to continued student drinking in the wake of school reform.

It is especially important for school administrators to address risky drinking behaviors among students who do not currently have a drinking problem. Research has shown that addictive disorders originate with risk factors that always include exposure to potential objects of addiction [27,50,52]. More specifically, repeated object exposure (i.e., alcohol consumption) can combine with an individual's underlying psychosocial and neurobiological vulnerabilities, resulting in desirable subjective shifts and the potential for developing an addictive disorder [53]. While each person has unique underlying vulnerabilities that make them more or less likely to develop an alcohol use disorder, reduction of opportunities to develop such a disorder (e.g., in the form of focused regulations) will benefit all students. With ever-increasing numbers of students entering four-year colleges in the U.S., clear explanations of institutional expectations and requirements regarding substance and behavioral addictions is an essential component of reducing such behaviors among students. For example, McDonnell (1994)[54] suggests that "alcohol education should be seen as part of the education of character" (p. 45). DFSCA provisions have been effective in stimulating alcohol education and policy development on college campuses; however, currently, some schools might not be providing reliable and accurate information about addictive behaviors, as evidenced by the inconsistent nature of the alcohol and gambling policies observed in this study. In the absence of a similar federal mandate requiring gambling education on college campuses, other drastic federal measures (e.g., the proposed ban on all collegiate sports betting in Nevada[55]) have been proposed to reduce student gambling. Such proposals are problematic because these efforts restrict the freedom of responsible gamblers and place policy pressure on states instead of schools, thereby diverting focus from those in need of effective regulation (i.e., college campuses). Scientific guidelines toward school regulation, similar to those proposed for federal regulation by OMB, will provide school administrators a solid foundation for creating comprehensive alcohol and gambling policies. It is the responsibility of administrators to make the first steps toward scientific selection of school policies and take substantive and definitive measures to increase addiction awareness and recovery among students.

### Caveats

Several methodological limitations to this study should be noted. First, this study relied solely upon written policy materials. Although we conducted an exhaustive search of the Internet and requested hard materials from schools, it is possible that we failed to identify some publicly available policy materials that could have provided additional information about schools in our sample. In addition, we identified our policy variables by examining existing policies and identifying relevant regulatory components; however, others attempting to replicate this study might identify and measure different aspects of policy and, consequently, obtain different results. Further, this study considered official alcohol policy content across a sample of U.S. colleges; however, assessing official policy provides no data on whether or how schools enforce their stated policies. It is possible, and in many cases even likely, that schools rely on informal rules and established precedents to govern alcohol use or gambling violations. Due to the complex nature of the relationships between school administrators, students, parents and legal authorities, many schools likely assess alcohol or gambling violations on a case-by-case basis; consequently, practice policy might be very different from the formal rules described by official school documentation.

### Opportunities for Future Research

This study has shown that there are many opportunities and perhaps an obligation to scientifically investigate the complex relationships between college alcohol and gambling policies and addictive behaviors. Future research, for example, should focus on policy enforcement and informal policies adopted by school administrators to provide a better understanding of current college practices regarding alcohol use and abuse. In addition, as schools begin to reevaluate and amend their substance abuse policies, longitudinal research could provide insight into the effects of policy revision on student behaviors. In addition, to advance our understanding of policy effects on intemperate patterns of behavior, future college-based research will need to examine the influence of policies on more specific behaviors. New research should deconstruct the macro indices (e.g., average rates) of binge drinking to determine whether policies can impact college violence, crime, driving under the influence, etc.

College alcohol and gambling policy data holds important potential for future research. For example, for students with alcohol or gambling related disorders attending colleges with policies that interfere with treatment or fail to support recovery, the rate of relapse is likely to be higher than under a more treatment favorable public policy context. Similarly, research can demonstrate that policies can influence the likelihood of early identification and intervention: under some unsympathetic regimes, students with addiction problems will not come forward for assistance or adhere to a prescribed treatment program. Finally, new research needs to show that without supportive policies to guide college staff responses to treatment seeking students, this population will miss both the college experience and the opportunity to build a healthy foundation for their future.

## Conclusions

In this study, we analyzed a representative sample of U.S. colleges to determine the attentiveness of school policies toward students with addictive disorders and their recovery. The results encourage the development and implementation of reporting tools (e.g., a rating system) that could prove valuable as both a resource for parents of at-risk students and a vehicle to raise public awareness. Identifying trends in collegiate policymaking as well as distinguishing strong and weak policies will allow us to begin to develop evidence based guidelines, or "best practices," upon which schools can base the development of future policy.

## Competing interests

The author(s) declare that they have no competing interests.

## Authors' contributions

HS conceived the study, was its principal architect, and was responsible for its overall conduct and exposition. AD was responsible for the data acquisition, adjudicated the reviewer ratings, and contributed to the drafting of the manuscript. RL was responsible for the statistical design, execution, and exposition. RK provided direct assistance to AD and participated in the preparation of the manuscript. DL contributed to the study structure, data analyses, and manuscript preparation. All the authors were members of the team that critically reviewed and coded the college policies. All authors read and approved the final manuscript.
